# Double marginalization: an ethnographic-ecological analysis of rural PE teachers’ professional development between urban and underdeveloped areas

**DOI:** 10.3389/fpubh.2025.1645977

**Published:** 2025-08-08

**Authors:** Jihong Yan, Xinyu Dai

**Affiliations:** ^1^Physical Education College, Jimei University, Xiamen, Fujian, China; ^2^Department of Physical Education, Xiamen University of Technology, Xiamen, Fujian, China

**Keywords:** rural physical education teachers, professional development, ecological-intersectional framework, educational equity, urban–rural divide, teacher agency

## Abstract

**Introduction:**

China’s rapid urbanization has exacerbated challenges in rural education, including resource disparities, shrinking student populations, and teacher shortages. Rural Health and Physical Education (HPE) teachers face acute professional development barriers, directly impacting their well-being and students’ physical health.

**Methods:**

This ethnographic study combines ecological and bioecological lenses to analyze rural HPE teachers’ professional development. Through three-stage sampling, 35 northeastern Chinese HPE teachers participated in semi-structured interviews and fieldwork. Data were thematically analyzed using an ecological-intersectional (E-I) framework across micro (individual), meso (interpersonal), exo (organizational), and macro (sociocultural) levels.

**Results:**

Findings demonstrate ecological interdependencies: Urbanization policies and unequal resource distribution created structural barriers, while school sports culture and support networks mediated outcomes. Teachers’ professional autonomy and rural commitment emerged as key adaptive factors.

**Conclusion:**

Multilevel interventions are proposed: macro policies (differentiated standards/resource compensation), organizational support (school leadership/home-school collaboration), and individual empowerment (autonomy/innovation training). This study advances the application of intersectional ecological theory in rural education contexts and offers actionable insights for equitable teacher policy development. It advances theoretical understanding of marginalized HPE teachers’ development while offering actionable policy insights with global relevance for underserved educational contexts.

## 1 Introduction

The professional development of rural Health and Physical Education (HPE) teachers is a critical foundation for teacher education policy design. Against the backdrop of China’s rapid urbanization, educational resource allocation exhibits pronounced urban–rural disparities. According to the latest statistics, China has 81,547 village-level schools, with an average enrollment of only 276 students per school. As urbanization progresses, this number is expected to decline further, exacerbating dual challenges of teacher shortages (1) and shrinking student populations. Notably, as a marginalized group within the education system, rural HPE teachers often experience a distinct form of “double marginalization”: as “commuter teachers” residing in cities but working in villages, they are perceived as “outsiders” by urban communities due to their rural workplace, while simultaneously being viewed as “urban outsiders” by the rural communities they serve. This unique positioning, coupled with the frequent marginalization of PE as a non-core subject, creates urgent needs for systemic research into their working conditions, career trajectories, and social support networks—particularly given the unique challenges and opportunities in physical education.

Existing studies have explored rural HPE teacher issues to some extent. Jiang et al. (1) found that while living subsidies temporarily enhance job attractiveness, they have limited long-term impact on career development and retention. Wang further identified three key dilemmas: excessive non-teaching burdens, lack of parental support, and low job satisfaction (2), aligning with Reid’s multidimensional rural development theory, which emphasizes holistic social, economic, and environmental perspectives (3). However, most research focuses on general-subject teachers, leaving rural HPE teachers understudied.

Harris’s research highlights that rural HPE teachers require multilevel support systems, with mentorship and financial aid being pivotal (4). Three teacher traits are particularly valuable: adaptability to leverage prior knowledge, professional preparedness, and resilience in under-resourced settings. Crucially, challenging negative stereotypes about rural life is essential for fostering professional identity—a finding offering valuable comparative insights.

Theoretical paradigms have also evolved. While early studies relied on single-factor analyses, multidimensional models like the Bioecological Theory of Human Development now provide systemic frameworks, locating teacher development within broader socio-ecological systems. For HPE teachers, professional growth encompasses not only pedagogical skills but also adaptation to and transformation of rural sports ecosystems.

Despite these advances, three gaps persist in China’s context: (1) insufficient ethnographic fieldwork to capture teachers’ lived realities; (2) neglect of developmental stage-specific needs; and (3) inadequate theorization of how intersecting power structures shape HPE teachers’ trajectories. These limitations obscure differentiated demands across career stages and hinder understanding of key influencing factors.

Emerging trends integrate theories like the Ecological–Intersectional (E-I) framework (5, 6), expanding analyses to structural inequities (e.g., institutional oppression) (7). This shift is significant: it transcends individualistic approaches, exposes structural roots of educational inequality, and offers new tools to examine rural HPE teachers’ struggles. Critically, our study introduces three paradigm-advancing innovations: First, we pioneer the application of the E-I framework to rural HPE teachers in the Global South—a departure from prior Western-centric coach studies. Second, we synthesize bioecological and intersectional lenses to reveal how *spatial marginalization* (urban–rural hierarchies) and *professional precarity* (non-core subject status) interactively constrain teacher agency. Third, we ethnographically document how these intersecting power structures manifest across China’s unique institutional landscape. Yet, empirical studies are needed to contextualize these theories within China’s rural education realities.

China’s unique complexities—left-behind children, resource constraints, and student health issues—heighten demands on HPE teachers. Studies confirm persistent barriers at micro (individual), meso (interpersonal), organizational, and macro (sociocultural) levels. This study thus addresses two core questions: (1) What is the current state of rural HPE teachers’ professional development? (2) Are personal traits or environmental factors more influential? Through typical case analysis, we spotlight teachers who overcome adversities to achieve senior titles or regional recognition. The findings will inform policy optimization and improve rural HPE quality, ultimately advancing teachers’ professional growth.

### 1.1 Ecological–intersectional model: research perspective

**Figure 1:**
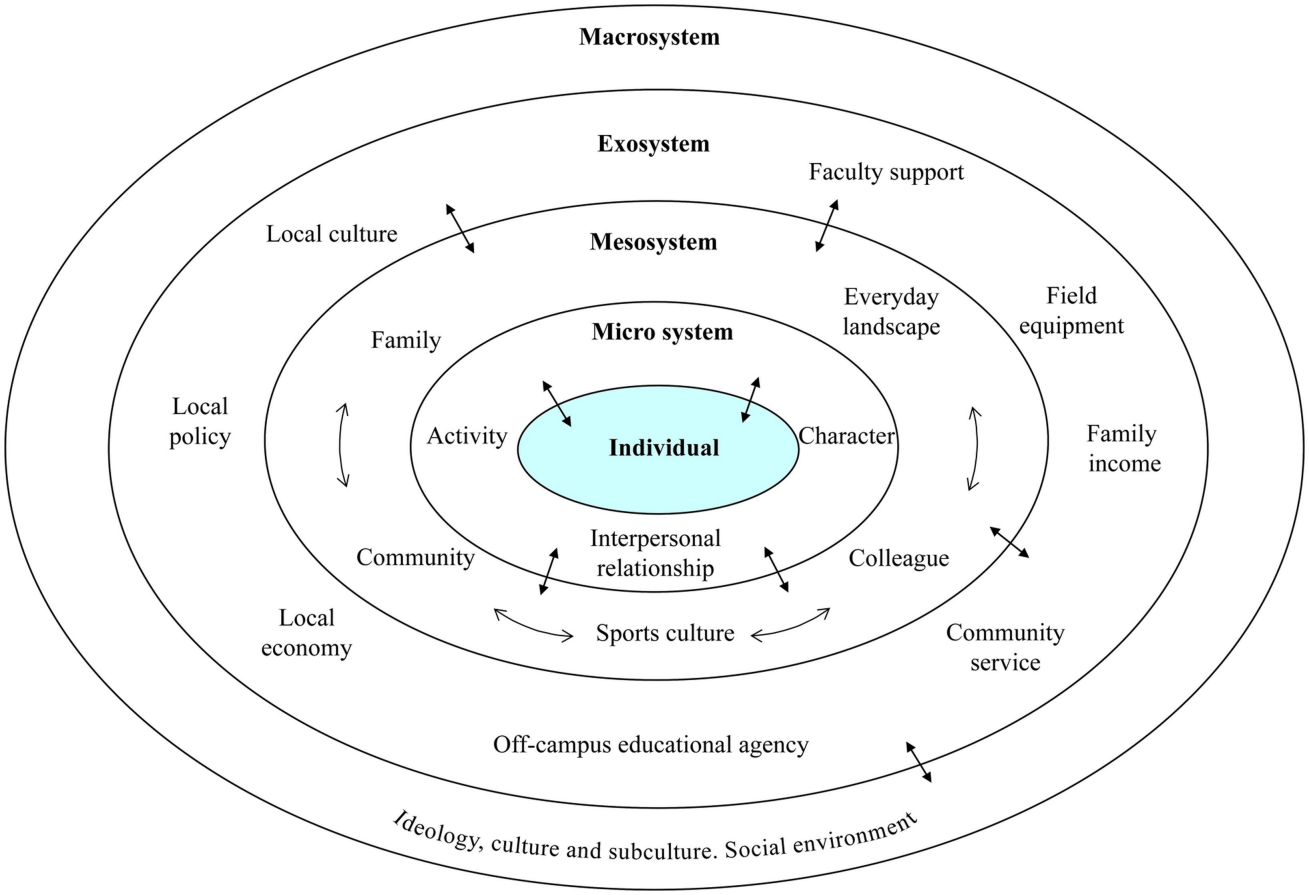
An ecological model of professional development for physical education teachers in rural primary and secondary schools.

Conducting research on the professional development of primary and secondary school physical education teachers through the dual lenses of ecology (8) and the Bioecological Theory of Human Development facilitates a paradigm shift from singular analytical frameworks toward a more comprehensive consideration of multidimensional factors and their dynamic interactions. This multidimensional approach aligns with the theoretical foundations of the E-I model (9), originally adapted from LaVoi and Dutove’s ecological framework, which has undergone significant theoretical refinement to incorporate three critical conceptual pillars: Bronfenbrenner’s ecological systems theory (10), intersectionality (11), and power (12). This evolved model delineates four interconnected socio-ecological levels that collectively shape professional experiences: the individual level (microsystem), interpersonal relationships (mesosystem), organizational contexts (exosystem), and broader socio-cultural influences (macrosystem), as illustrated in Figure 1. LaVoi particularly emphasizes that the integration of intersectionality theory within the E-I model compels researchers to examine physical education teachers’ professional trajectories through multiple identity dimensions, including their lived realities, available social support systems, and institutional environments. While much of the existing evidence underpinning this framework derives from studies on professional coaches, its adaptation to analyze systemic inequities in rural education—particularly through the novel integration of Chinese spatial hierarchies and professional precarity—represents a significant theoretical advancement. Its theoretical robustness and multidimensional architecture make it particularly suitable for guiding the interview design and data analysis processes in our investigation focusing on rural school physical education teachers’ professional development.

The first and most proximal layer is the individual/intrinsic level, encompassing biological and psychological factors such as motor skills, cognition, emotions, beliefs, and values—primarily reflecting the educational dedication and professional knowledge/abilities of primary and secondary HPE teachers. For example, a typical barrier at this level is value cognition bias, where some rural HPE teachers attribute professional development challenges to external factors like “insufficient facilities” while overlooking the critical role of endogenous variables such as their own teaching innovation capabilities.

The next layer is the microsystem, consisting of environments where teachers engage in direct, face-to-face interactions. Its core components include teaching activity settings and role-relationship networks. This system influences teachers’ professional behaviors through an “experience-feedback” loop mechanism—for instance, when teachers consistently receive positive student responses, their willingness for pedagogical innovation is reinforced.

The third layer is the mesosystem, defined by family, colleagues, work environments, and school sports culture. It can be categorized into three dimensions: space, time, and culture. Spatially, family demands (e.g., childcare responsibilities) may conflict with job requirements; temporally, professional development (e.g., training) competes with daily teaching for resources; culturally, the marginalization of PE as a “non-core subject” clashes with quality education expectations. Such cross-pressures may lead teachers to adopt “strategic compromises,” such as overemphasizing fitness-test drills to meet administrative evaluations.

The fourth layer is the exosystem, the most distal layer, encompassing regional socio-cultural and economic development factors—normative and cultural systems that indirectly influence teachers. Though not directly affecting practitioners, these macro-factors impose tangible constraints through organizational decisions (e.g., school budget allocations). For instance, compared to urban areas, rural schools often lack adequate resources, from high-tech multimedia tools to basic sports equipment (e.g., balls, gymnastics apparatus), potentially undermining teaching quality and hindering professional development.

## 2 Methodology

### 2.1 Participants

Prior to recruitment and data collection, institutional ethical approval and informed consent (including procedures) were obtained. Specifically for online interviews: (1) recruitment invitations explicitly stated research objectives and data usage protocols; (2) written digital consent was secured via secure messaging application before each session; (3) participants received real-time control over audio/video sharing. To enhance participant diversity, a three-stage sampling strategy was employed to ensure theoretical saturation while improving sample representativeness.

Stage 1 (August–October 2024) used open sampling to select seven rural HPE teachers from three northeastern provinces (Liaoning, Jilin, Heilongjiang) for semi-structured interviews, focusing on their professional development status and influencing factors. Sample selection considered variables such as provincial distribution and school type, forming the preliminary research framework.

Stage 2 (October–December 2024) applied purposive sampling, conducting field observations in 10 rural schools and using snowball sampling to access key informants (13). This approach effectively addressed challenges in recruiting sensitive-topic participants, adding 18 teachers to the interview pool.

Stage 3 employed theoretical sampling, selectively interviewing 10 teachers with typical characteristics (varying in age, gender, teaching experience, and professional rank) based on emerging conceptual categories until theoretical saturation was achieved. The final sample comprised 35 participants (see Table 1), with demographic characteristics fully covering all required research dimensions.

**Table 1:** Characteristics of the interviewed teachers.

Participant	Age	Gender	Length of service in teaching	Educational background
LJK	47	Male	27	Bachelor
JJL	35	Female	12	Bachelor
HQK	28	Male	4	Bachelor
LWD-04	37	Male	11	Bachelor
Wei Y	30	Male	7	Bachelor
Yang X	57	Male	34	Bachelor
Li CH	28	Female	3	Bachelor
Li J	51	Male	28	Bachelor
Mao JL	28	Male	6	Bachelor
Lin Y	31	Male	8	Bachelor
Wei JF	55	Male	35	Bachelor
Wang XX	30	Male	2	Bachelor
Gao XW	52	Male	29	Master
Shang K	29	Female	2	Bachelor
Yao ZH	27	Female	3	Bachelor
He XX	53	Male	30	Bachelor
Huo XL	28	Male	4	Bachelor
Gao XX	28	Male	3	Bachelor
Tian ZJ	33	Male	7	Bachelor
Zheng Y	36	Male	10	Bachelor
Zhang SY	33	Male	8	Bachelor
Gao Y	34	Male	9	Bachelor
Shu JL	43	Female	16	Bachelor
Liu XM	50	Male	27	Bachelor
Lin XL	35	Male	8	Master
Wen S	30	Female	4	Bachelor
Zhang ZY	30	Male	6	Bachelor
Jiang CD	34	Female	10	Bachelor
Li Y	38	Male	14	Bachelor
Wei BT	42	Male	20	Master
Liao XP	55	Male	32	Master
Shi XG	50	Male	28	Master
Cui LP	53	Male	30	Master
Ji L	49	Male	29	Master
Wu Q	56	Male	30	Master

### 2.2 Data collection

This study employed semi-structured interviews to collect data, completing a total of 35 interviews (6 face-to-face and 29 conducted online via Microsoft Teams), each lasting 45–90 min. The interviews covered demographic characteristics, factors influencing professional development, job satisfaction, and career planning.

The interview process strictly adhered to qualitative research ethics: First, informed consent was obtained, with the research purpose clearly explained and confidentiality agreements signed. Second, face-to-face interviews were conducted in quiet, comfortable settings with audio recording, while online interviews were scheduled during participants’ free time. To address potential information loss in transcriptions, a secondary verification mechanism was established, with follow-up clarifications via secure messaging application platforms.

In terms of interview techniques, the researchers: (1) used guiding questions to focus on rural HPE teachers’ professional development; (2) practiced active listening and provided non-verbal feedback when appropriate; (3) probed at critical points; and (4) allowed participants to skip sensitive questions. At the end of each interview, participants were invited to add further comments to ensure data completeness.

Data processing followed verbatim transcription principles, supplemented by triangulated observational field notes (e.g., teacher pedagogical practices, school sports facilities utilization) and institutional data analysis (e.g., local policy documents, teacher promotion timelines). A triple-checking mechanism ensured accuracy: cross-verification between original recordings/observational notes and transcripts, member-checking by participants, and review by the research team. All materials (interviews, field notes, institutional records) were archived according to standardized protocols to ensure traceability. For thematic analysis aligned with the E-I framework, themes were initially identified by the first author and then rigorously validated through structured discussion and consensus-building within the full research team; inter-coder reliability was formally assessed on a 20% sample, achieving near-perfect agreement (Cohen’s Kappa = 0.92), confirming theme robustness. This comprehensive methodological design laid a reliable foundation for subsequent analysis.

### 2.3 Data analysis

**Figure 2:**
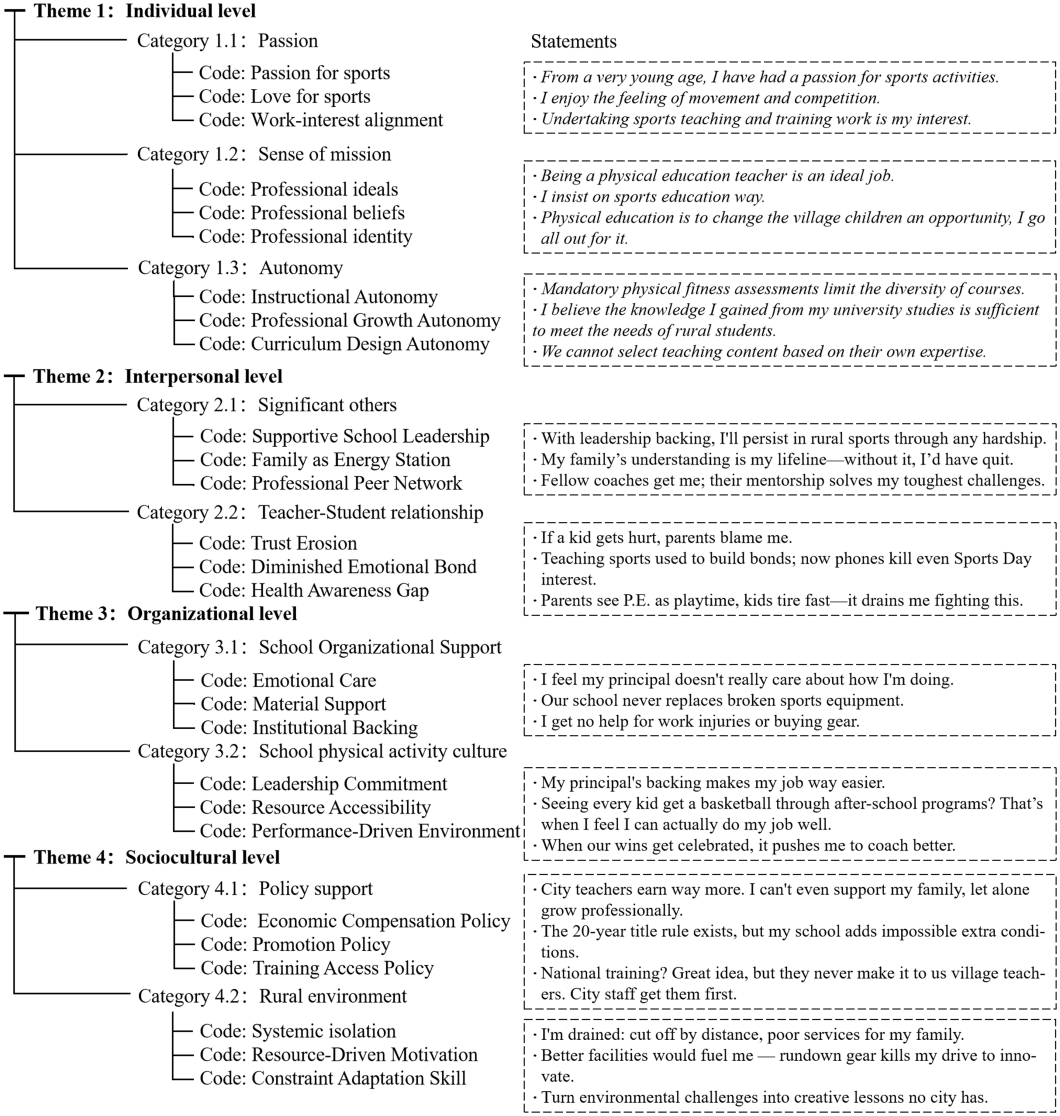
Hierarchical coding framework.

This study applied thematic analysis to systematically identify data patterns, a method well-suited for examining complex phenomena such as teachers’ professional learning (14). The analysis followed a three-stage process (Figure 2): First, preliminary coding frameworks were developed through concurrent analysis of field notes during fieldwork. Next, two rounds of iterative coding refined core themes to ensure analytical depth (15). Finally, the Ecological–Intersectional Model was introduced as a theoretical lens to systematically examine the multidimensional interactions shaping professional development. To enhance validity, data triangulation integrated interviews, observations, and documentary materials (16), with regular coding reviews conducted by three researchers. Study credibility was ensured through: (1) establishing traceable analytical trails; (2) providing thick descriptions of raw data; and (3) explicitly defining transferability conditions regarding research boundaries (17).

### 2.4 Ethics approval

Ethics approval for this study was obtained from Capital university of physical education and sports Research Ethics Committee (No. 2025A015). All participants signed an informed consent form before participating in the study. Participants had to confirm their informed consent before proceeding with the investigation. Verbal consent to participate in the interview was obtained at the beginning of each session. To ensure privacy and confidentiality, all data were anonymized and stored securely.

## 3 Results

### 3.1 Individual level

#### 3.1.1 Sustaining passion and mission in rural physical education

Professional sentiment plays a pivotal role in the professional development of rural HPE teachers. Generally, professional sentiment is composed of factors such as professional ideals, professional beliefs, and professional identity. Among these, professional ideals represent the inner aspirations of rural teachers—envisioned future levels of professional development shaped by societal expectations, industry standards, and personal knowledge and abilities. These ideals manifest as scientifically grounded professional development goals and plans, providing motivational support for their growth. Professional beliefs serve as the behavioral guidelines for rural teachers’ practice, gradually formed during their professional growth, primarily reflecting their recognition of unique perspectives or values in educational endeavors. Professional identity refers to rural HPE teachers’ profound awareness of their professional roles, characteristics, and value. Those with strong professional identity typically maintain an enthusiastic work ethic and exhibit a spirit of innovation.

Notably, most rural HPE teachers in China live in cities but work in rural areas, leading to a dual marginalization: they are seen as outsiders by urban populations and as “non-locals” by villagers. Caught between urban and underdeveloped regions, along with the trend of urbanization, they find themselves adrift, disconnected from both rural and urban communities. As a result, many lose their passion for advancing rural education, causing their professional development to stagnate. However, some teachers reside and work in rural areas. For instance, Teacher Huo, throughout his eight-year teaching career, has maintained a strong drive for excellence. As a normal university graduate who grew up in the countryside, he harbors a deep sentiment for rural life and PE teaching. As Huo reflected:

*“I’m very satisfied with teaching football. I was born and lived in the countryside for over a decade, so I have a strong passion for sports. I’ve always loved playing football, and now I get to teach it—it feels like my work and interests are perfectly aligned. Especially when leading students to competitions, I’m not just their teacher or coach but more like an older brother, accompanying them as they grow.” (Huo, interview).*

The study found that most rural HPE teachers chose their specialization in high school due to their athletic skills or love for sports, resulting in a high level of professional identity. This strong identification significantly fuels their professional development.

#### 3.1.2 The autonomy dilemma in rural HPE teaching

All humans possess an innate psychological need for autonomy (18). However, motivation is only activated, sustained, or enhanced when this need is perceived to be fulfilled. Contrary to expectations, survey findings indicate that most respondents considered autonomy to have a relatively weak impact on their professional development (19). This discrepancy may be linked to the challenges faced by HPE teachers in China. For instance, rural HPE teachers currently demonstrate low awareness of the importance of cultivating personal autonomy, which directly correlates with their weak professional autonomy during career development.

Among recent HPE teacher graduates and newly hired rural teachers, the primary focus remains on personal quality of life, with most exhibiting minimal awareness of autonomous professional growth. As for experienced teachers, while their long tenure grants them familiarity with rural educational contexts, teaching methods, and extracurricular activities—enabling them to competently deliver sports and health education—they often invest less time in learning new knowledge in the later stages of their careers. Although proficient in various sports techniques and games, prolonged engagement in monotonous teaching activities leads to complacency. Moreover, mandatory physical fitness assessments (e.g., dedicating class time to endurance running or sit-up drills for standardized tests) imposed by provincial or municipal authorities further constrain their instructional autonomy. Consequently, sustaining high levels of professional autonomy proves challenging, particularly evident in their underdeveloped learning, reflective, and research capacities.

Our findings suggest that rural HPE teachers’ autonomous awareness in professional development requires enhancement. Policymakers should reduce the weight of physical fitness tests in student evaluations and instead promote autonomy by expanding opportunities for curriculum design, creating courses aligned with teachers’ strengths, and fostering a more supportive professional environment.

### 3.2 Interpersonal level

#### 3.2.1 Significant others in rural HPE teachers’ professional development

The concept of “significant others,” initially proposed by American sociologist C. Wright Mills in the mid-19th century (20), refers to individuals who exert profound influence during a person’s socialization process (21).

Throughout various career stages of rural physical education teachers, these significant others play pivotal roles in shaping professional identity and facilitating role transitions. With their guidance, support, and encouragement, rural HPE teachers demonstrate marked improvement in professional development. Our research reveals that significant others in this context are remarkably diverse, including regional teaching researchers, members of master teacher studios, local peers encountered during competitions or training sessions, higher education experts, and distinguished teachers from other schools. Notably, sports activities—particularly ball games—serve as primary platforms for HPE teachers to interact with school administrators, fostering friendships that encourage open communication and mutual understanding.

As evidenced by field observations:

Teachers Wei and Yang disclosed that despite having opportunities to transfer to urban schools, they chose to remain in rural institutions due to harmonious relationships with school leadership. They expressed particular appreciation for administrators who actively supported physical education, noting that they would likely stay until their principal’s retirement. This testimony underscores the unique value of school administrators—especially principals—in facilitating rural HPE teachers’ professional growth (Fieldnote, 23/03/2022).

Furthermore, family support—particularly from spouses and parents—emerges as another crucial factor sustaining teachers’ commitment to rural education. Multiple interviewees emphasized that their enduring dedication to challenging rural postings stemmed from family members’ understanding and emotional sustenance, describing their families as “energy stations” that continuously fuel professional advancement. Through sustained interactions with these significant others, rural HPE teachers receive indispensable encouragement and support that guides their progressive development.

#### 3.2.2 Teacher-student relationship challenges in rural physical education

A positive teacher-student relationship was associated with enhanced emotional engagement, increased sports motivation, and better academic performance in physical education (22). Primary and secondary school physical education teachers in rural areas generally perceive their students as innocent, simple, frugal, and energetic, finding joy in engaging with them through games and competitions. However, with the advancement of digital technology, these left-behind children are increasingly misled by online misinformation or succumb to digital addiction, including excessive mobile phone use. Decades ago, rural children exhibited superior physical fitness compared to their urban counterparts, as they actively played on country paths, climbed trees, and swam in natural settings. Yet, the decline in these traditional physical activities, coupled with the negative impacts of technology overuse, has led to a noticeable deterioration in rural students’ physical conditioning, now even falling below urban standards. This weakened physical capacity renders them less capable of handling moderate to high exercise loads during PE classes. Compounding these challenges, the erosion of the once-pristine teacher-student relationship has been exacerbated by shifting societal attitudes—students are no longer as simple and trusting as before, and teachers face increasing accountability, even legal liability, when injuries occur during class. This climate of distrust not only discourages educators from organizing physically demanding activities but also contributes to professional disillusionment, as teachers feel undervalued and unfairly scrutinized. Furthermore, the pervasive lack of health awareness among rural students and their families amplifies these issues, creating a cycle of declining engagement in physical education and stagnating professional development for teachers in already under-resourced rural schools.

### 3.3 Organizational level

#### 3.3.1 Organizational support deficits in rural HPE teacher development

Organizational Support refers to teachers’ perceived level of institutional valuation of their contributions and concern for their well-being (23). As a critical organizational-level resource, this perceived support has been empirically demonstrated to significantly enhance teachers’ work attitudes and behavioral performance, with particularly notable effects in mitigating occupational burnout (24, 25).

Of special significance is the consensus that organizational support constitutes the most decisive factor influencing the professional development of rural HPE teachers. Specifically, this support system encompasses three dimensions: emotional care (e.g., leadership concern), material support (e.g., teaching equipment), and institutional backing (e.g., welfare policies). Research indicates that workplace support from colleagues and supervisors exhibits a more pronounced negative correlation with emotional exhaustion compared to family support. Given the universality of this phenomenon among both urban and rural educators, this study will focus on the unique challenges within rural school HPE teaching contexts.

Field investigation data reveal three salient issues: First, severe infrastructure deficiencies manifest as high rates of hardened sports surfaces and prolonged equipment replacement cycles. Second, professional marginalization is evident through HPE teachers’ disadvantaged status in professional title evaluations and disciplinary decision-making. Third, constrained professional capabilities emerge due to objective conditions limiting effective application of specialized motor skills, such as basketball tactical training.

Beyond inadequate teaching resources, flawed welfare systems present major obstacles: most respondents reported lacking subsidies for sports equipment purchases and inadequate medical coverage for work-related injuries. Regarding Workload-Induced Stress, despite societal advocacy for flat management structures and workload reduction, rural HPE teachers widely perceive that excessive workloads adversely affect their professional development. The reality reflects three contradictions: policy implementation (extracurricular activities increasing teaching loads), staffing constraints (most teachers assuming concurrent administrative roles), and professional development (only 1–2 teachers per school receiving regular training opportunities). These multidimensional pressures ultimately trap professional development in a “high-load, low-growth” vicious cycle.

#### 3.3.2 School physical activity culture as a catalyst for HPE teacher development

The diversification of sports culture, encompassing activities such as yoga, hiking, and skateboarding, has been accompanied by increased public participation in various physical activities (26). However, rural students often face significant barriers in accessing these emerging sports due to limited exposure and financial constraints in purchasing necessary equipment or receiving professional training.

Current research indicates that rural physical education teachers widely recognize the substantial impact of school sports culture on professional development. Field investigations reveal that school leadership’s commitment to fostering a sports-oriented learning environment, as well as a school’s designation as a specialized institution for particular sports, significantly influences both classroom instruction and extracurricular training. Many motivated frontline HPE teachers report that working in sports-focused schools enables them to effectively utilize their specialized skills. This trend has become particularly noticeable in recent years with increased national emphasis on physical education, leading to greater administrative support where “athletic achievements are viewed as core indicators of school competitiveness.” The establishment of regular regional sports events has contributed to enhancing student fitness and enriching campus culture.

Within such vibrant sports environments, HPE teachers develop comprehensive professional capabilities through coaching and instructional practice. While there exists a tendency to prioritize a select group of “talented” or “gifted” students – potentially leading to greater emphasis on competition over comprehensive instruction (27) – this dynamic nonetheless motivates teachers to proactively acquire coaching and refereeing qualifications, ultimately improving their pedagogical and training methodologies, which positively impacts their professional growth. As Teacher Jiang reflected:

*“Our school’s basketball team ranked fifth in the provincial tournament. Through extracurricular programs, we ensure every student has access to basketball equipment. This sports-friendly atmosphere significantly facilitates our teaching efforts and reinforces my sense of professional fulfillment, leading to noticeable improvements in both classroom instruction and training capabilities” (Jiang, interview).*

Conversely, HPE teachers working in rural schools with underdeveloped sports cultures frequently demonstrate passive attitudes and minimal professional engagement. Moreover, a robust school sports culture can extend physical education beyond traditional school hours into broader community spaces (28), playing a pivotal role in the ecosystem of HPE teachers’ professional development. This warrants focused attention from relevant stakeholders. It should be noted that school sports culture represents a qualitative, intangible factor that cannot be rapidly transformed through curriculum standards or reforms alone. Its optimal function in promoting teacher development requires coordinated efforts at national, societal, and institutional levels.

### 3.4 Sociocultural level

#### 3.4.1 Policy support gaps in rural HPE teachers’ professional development

From the perspective of policy implementation, although the Chinese government has introduced a series of policies in recent years regarding the appointment, training, recruitment, and compensation of rural physical education teachers, field research reveals a significant gap between policy outcomes and expected objectives (29). This discrepancy manifests in several aspects: delayed policy dissemination (e.g., untimely receipt of training notifications), absence of implementation monitoring mechanisms, and failure to effectively enhance teachers’ professional status. Such policy implementation deviations have trapped rural physical education teachers in a triple dilemma—inadequate economic compensation, insufficient social recognition, and limited access to educational resources—thereby severely constraining their professional development.

International comparative studies further corroborate the critical impact of economic compensation. Banerjee and Duflo (30) found that a 40% increase in teacher salaries in the Santiago region of Chile directly led to a 17–21 percentage point reduction in turnover rates. In contrast, rural teachers in northeastern China have long suffered from low compensation levels. For instance, Teacher S, one of the interviewees, receives only 50% of the salary of urban teachers in the same region. The economic pressure was poignantly articulated by Teacher Li during the interview:

*“I now have a family and children. The main reason I work is to make a living. If the income from this job cannot even feed myself and my family, how can I dedicate myself to others, let alone pursue professional development?” (Li, interview).*

Regarding professional title evaluation policies, although the three northeastern provinces (Liaoning, Jilin, and Heilongjiang) have implemented differentiated preferential policies for rural teachers—such as allowing those with 20 years of teaching experience to be directly promoted to senior professional titles without assessment—these policy benefits are significantly diluted at the school implementation level. Specific manifestations include: (1) quota limitations leading to distorted competition (e.g., some schools in Liaoning Province adding extra requirements like “winning municipal-level excellence in teaching within 3 years”); and (2) structural deficiencies in rural teachers’ access to high-quality teaching demonstration opportunities (e.g., scarce municipal-level evaluation opportunities). This disconnection between policy design and implementation has transformed what was intended to be a professional development incentive into a career barrier for rural physical education teachers.

In terms of training policy implementation, the research found that rural physical education teachers have limited access to national-level training opportunities. Additionally, their overall welfare benefits are comparatively inferior to those of urban teachers. Due to inadequate implementation of rural physical education policies and an incomplete supporting system, the direct impact of these policies on the professional development of rural physical education teachers remains suboptimal.

#### 3.4.2 Rural environment as a double-edged sword for HPE teachers’ professional development

Research findings indicate that, from the perspective of rural environments, the majority of physical education teachers interviewed believe that the objective constraints of rural settings significantly impact their professional development. Specifically: ① In terms of living convenience, the remote geographical location of rural areas creates systemic challenges for teachers in daily commuting (31), attending external training programs, accessing quality education for their children, and obtaining adequate healthcare resources; ② Regarding teaching conditions, sports facilities and equipment in village schools generally fall below urban standards. This contrasts with Ma HM’s findings which noted rural teachers’ salaries exceeding urban counterparts by 6.72–8.44% (32), while our investigation in Northeast China revealed the opposite trend, potentially reflecting regional economic disparities. This dual disadvantage of environment and resources creates persistent conflict between teachers’ expectations and actual working conditions, thereby diminishing their professional development efficacy.

However, in-depth analysis reveals a distinct bidirectional characteristic in how rural environments influence teacher development. Some teachers demonstrate resilience and growth through professional identity adaptation: As interviewee Guo described, “We often have to clear snow for classes in winter, but by developing activities like snow football and homemade curling equipment, we have created unique teaching content.” Such adaptive professional practices show that environmental constraints can, depending on individual characteristics, transform into catalysts for pedagogical innovation.

In striking contrast, another group exhibits an environment-sensitive development pattern. Their professional growth pace shows significant positive correlation with material conditions (equipment, facilities, etc.), making them prone to burnout when infrastructure fails to meet expectations. This divergence reveals a dual structure in current rural teacher composition: ① Passive selectors (those entering underdeveloped areas due to limited professional competitiveness); ② Active contributors (motivated by altruism). Notably, the latter group represents a limited proportion in our sample, yet their cases of transcending environmental constraints provide crucial insights for optimizing rural teacher policies.

## 4 Discussion

This study reveals that the professional development of rural physical education (PE) teachers in China is shaped by the complex interplay of ecological factors across individual, interpersonal, organizational, and socio-cultural dimensions. Our findings extend the Ecological–Intersectional (E-I) framework by demonstrating how structural marginalization (e.g., urban–rural hierarchies, PE subject precarity) interacts with teacher agency to produce divergent developmental trajectories—a nuance underexplored in prior applications of bioecological theory to educational contexts.

At the individual level, professional commitment serves as the core driver, though most teachers experience developmental stagnation due to their dual marginalized status. While Harris (33) identified financial incentives as primary motivators for Western rural teachers, our participants demonstrated that professional passion and mission can sustain commitment even amidst material disadvantages—exemplified by deeply rooted educators like Teacher Huo whose intrinsic motivation aligns with Sun’s findings (34) on Chinese rural teacher resilience. The prevalent lack of professional autonomy stems from policy constraints, career stage variations, and environmental limitations.

Interpersonally, significant others alleviate professional isolation through emotional and resource support. However, teacher-student relationships deteriorate due to the digital divide and eroding trust, a phenomenon intensified by left-behind children’s internet addiction and technology-driven distraction in physical activities (35). This creates a vicious cycle of “low exercise engagement-limited skill development.

Organizationally, inadequate institutional support and excessive workloads emerge as critical barriers. Despite national policies mandating salary subsidies for underdeveloped areas, our Northeast China sample revealed reversed economic trends—highlighting how regional disparities produce contradictory outcomes under uniform compensation frameworks. While school sports culture fosters competency growth through competitive mechanisms (as in Teacher Jiang’s case), its extended educational potential within communities remains underutilized.

### 4.1 Comparison with prior research

Our socio-cultural findings reveal significant tensions between policy design and implementation. Where Vegas demonstrated salary increases reducing teacher turnover (36), China’s promotion policies often fail due to quota distortions—exposing a policy-implementation gap evidenced in resource allocation conflicts between cities and villages (37). This gap resonates with the broader Global South challenge of policy execution under pressure, as seen in the unprepared transition to online education during COVID-19 in Pakistan (38, 39), yet is uniquely shaped in China by the entrenched urban–rural dual structure. Furthermore, environmental impacts demonstrate a duality: passively recruited teachers face burnout risks, while devoted educators transform constraints into innovation (e.g., snow football curricula), a pattern aligned with the emotional intelligence-mediated agency model and cultural capital stratification (38, 39). Importantly, our study reveals that this transformation often occurs alongside significant, gendered pressures. Similar to the way the pandemic reinforced traditional wifehood and motherhood expectations for educated women in Pakistan, compressing their professional identity space, female rural HPE teachers in China frequently navigate compounded burdens of professional marginalization and intensified familial responsibilities within socio-cultural norms.

Our findings refine ecological-intersectional theory by demonstrating how rural PE teachers navigate intersecting pressures of urban–rural divides, subject marginalization, gendered expectations, and policy gaps. Specifically, we: (1) expand the E-I framework to recognize policy-implementation disparities as systemic barriers at the macrosystem level; (2) reveal environmental duality—where constraints like snow clearance become catalysts for pedagogical innovation (e.g., snow football)—challenging bioecological models’ stressor-centric assumptions; and (3) propose a “pressure-innovation” dynamic that redefines teacher agency in marginalized Global South contexts, highlighting unique strategies (e.g., local curriculum development, informal networks) employed by Chinese rural HPE teachers to circumvent barriers that parallel, yet differ from, the technical and personal obstacles faced by educators in contexts like Pakistan’s shift to online learning.

Essentially, rural PE teachers’ professional development represents an ongoing negotiation between constraining factors (policy-environment-resources) and enabling factors (passion-innovation-support networks). Breaking the “high-pressure/low-growth” paradox requires multi-tiered interventions: differentiated policy compensation (salary/promotion reforms), optimized organizational support (workload reduction/facility guarantees), and cultivated individual resilience—particularly through mechanisms that transform environmental constraints into professional opportunities, as demonstrated by our adaptive cases.

## 5 Conclusion

Based on an ethnographic study of 35 rural primary and secondary school physical education teachers in China, this research unveils the complex mechanisms underlying the professional development of this doubly marginalized group within the urban–rural dual structure. The findings demonstrate that rural HPE teachers’ professional development exhibits distinctive ecological interactions: at the exosystem level, policy orientations and uneven resource allocation during urbanization constitute structural constraints; at the mesosystem level, school sports culture and colleague/family support networks serve as crucial mediating variables; while at the microsystem level, teachers’ intrinsic commitment and professional autonomy play significant moderating roles. This discovery provides empirical evidence for understanding “agentic practices in marginalized contexts.”

The study particularly highlights that rural HPE teachers’ double marginalization manifests both in institutional disparities of urban–rural resource distribution and societal cognitive biases regarding subject status. Within such structural constraints, highly autonomous teachers employ three strategies to achieve breakthroughs: (1) transforming environmental limitations into distinctive teaching content (e.g., developing local sports curricula); (2) establishing informal professional learning networks (e.g., cross-school teaching research communities); (3) proactively securing policy resources (e.g., collaborating with community sports organizations). These findings corroborate the pivotal role of “proximal processes” in bioecological theory while enriching international discourse on marginalized teachers’ agency.

While offering valuable insights, this study acknowledges certain limitations. The ethnographic focus, primarily on 35 teachers within specific geographic regions, limits the broad generalizability of findings. Potential response biases inherent in qualitative research and the absence of perspectives from key stakeholders like students or school leaders represent further constraints that future work should address. Evidence-based policy recommendations encompass three dimensions: At the macro level, establishing urban–rural differentiated HPE teacher development standards with emphasis on compensatory policies like commuting subsidies and regional allowances; At the meso level, schools should enhance leadership support and foster equitable working environments, promoting sports’ benefits for physical/mental health and academic performance through health seminars to reshape parental attitudes, thereby forming family-student-parent collaborative support networks; At the micro level, cultivating teachers’ rural commitment and professional autonomy should be prioritized, safeguarding their curricular decision-making power while enhancing their capacity to transform constraints into pedagogical innovations through case-based training. Future research should further investigate the dynamic alignment between policy interventions and teacher development trajectories through longitudinal studies tracking career progression, particularly focusing on critical transition points for those overcoming ‘double marginalization’. Additionally, comparative studies examining regional variations in rural HPE teacher experiences and support mechanisms would significantly deepen our understanding.

## Data Availability

The raw data supporting the conclusions of this article will be made available by the authors, without undue reservation.
